# Supplementary material for: From surviving to thriving: How sleep, physical activity, and diet shape well-being in young adults
Source: PLoS One. 2025 Aug 27;20(8):e0329689. doi: 10.1371/journal.pone.0329689 (PMC12385438; doi:10.1371/journal.pone.0329689)
Supplement: S1 File — S1 Table. Bivariate correlations among the health behaviors. *p < .05, **p < .01, ***p < .001; F = Fitbit measured; FV = fruit and vegetable consumption; UPF consumption = ultra-processed foods (sweets and chips combined); Correlations above diagonal=between-person, below diagonal=within-person. Sleep quality measures are those used in each study’s respective final model (i.e., Study 1 and 3 = T scores, Study 2 = raw scores). Sleep quality values have been inversed for Study 1 and 3 so that higher values indicate greater sleep quality. S2 Table. Bivariate correlations between covariates and health behaviors (between-person). *p < .05, **p < .01, ***p < .001; BMI = body mass index; F = Fitbit measured; Raw = raw sleep quality scores; SES = socioeconomic status; T = sleep quality T scores. 1 male (0) vs. female/gender diverse (1). 2NZ European (0) vs non-NZ-European/mixed ethnicity (1). 3raw scores on depressive measures. Sleep quality values have been inversed for Study 1 and 3 so that higher values indicate greater sleep quality. S3 Table. Intra-class coefficient (ICC) values for the health behaviors and well-being measures in the daily diary studies. FV = fruit and vegetables; ICC = intra-class correlation coefficient reflecting the percentage of variance attributable to between-person differences; SE = standard error; UPF = ultra-processed foods. The ICC represents the variance attributable to between-person differences (ICCs > .5 indicate more variance is between people; ICCs < .5 indicate more variance is within-people). Daily well-being was measured using the Flourishing Scale in Study 2 and the WHO-5 Well-Being Scale in Study 3 both adapted to daily reporting. S4 Table. A table comparing the outputs from Study 3’s models using the self-report measure of physical activity and Fitbit device measured physical activity. AIC = Aikake’s Information Criterion; BIC = Bayesian Information Criterion; FV = fruit and vegetable consumption; Quad = quadratic term; SES = soc [file pone.0329689.s001.docx]

SI Table 1. Bivariate correlations among the health behaviors.

| Variables | (1) | (2) | (3) | (4) | (5) |
| --- | --- | --- | --- | --- | --- |
| Study 1 |  |  |  |  |  |
| (1) Sleep quality | - | 0.021 | 0.067^*^ | -0.153^***^ |  |
| (2) Physical activity |  | - | 0.420^***^ | 0.276^***^ |  |
| (3) FV consumption |  |  | - | 0.168^***^ |  |
| (4) UPF consumption |  |  |  | - |  |
| Study 2 |  |  |  |  |  |
| (1) Sleep quality | - | 0.158^***^ | 0.170^***^ | -0.136^***^ |  |
| (2) Physical activity | 0.090^***^ | - | 0.254^***^ | -0.001 |  |
| (3) FV consumption | 0.052^***^ | 0.059^***^ | - | -0.033 |  |
| (4) UPF consumption | 0.028^**^ | -0.011 | -0.042^***^ | - |  |
| Study 3 |  |  |  |  |  |
| (1) Sleep quality | - | 0.129 | 0.080 | 0.209^**^ | -0.117 |
| (2) Physical activity | -0.079^*^ | - | 0.255^***^ | 0.149^*^ | 0.017 |
| (3) Physical activity (F) | -0.042 | 0.382^***^ | - | -0.024 | -0.040 |
| (4) FV consumption | 0.005 | 0.067^*^ | -0.001 | - | 0.046 |
| (5) UPF consumption | 0.043 | 0.016 | 0.050 | -0.046 | - |

*p<.05, **p<.01, ***p<.001; F=Fitbit measured; FV= fruit and vegetable consumption; UPF consumption= ultra-processed foods (sweets and chips combined); Correlations above diagonal=between-person, below diagonal=within-person. Sleep quality measures are those used in each study’s respective final model (i.e. Study 1 and 3=T scores, Study 2=raw scores). Sleep quality values have been inversed for Study 1 and 3 so that higher values indicate greater sleep quality.

**SI Table 2***.* **Bivariate correlations between covariates and health behaviors (between-person).**

| Variables | Age | Gender^1^ | Ethnicity^2^ | BMI | SES (child) | Depressive symptoms^3^ |
| --- | --- | --- | --- | --- | --- | --- |
| Study 1 |  |  |  |  |  |  |
| (1) Sleep quality, Raw | 0.060 | -0.096^*^ | 0.065^*^ | -0.071^*^ | 0.147^***^ | -0.476^***^ |
| (2) Sleep quality, T | 0.071^*^ | -0.094^*^ | 0.057 | -0.067^*^ | 0.146^***^ | -0.476^***^ |
| (3) Physical activity | 0.185^***^ | -0.104^***^ | -0.067^*^ | -0.042 | 0.220^***^ | 0.106^***^ |
| (4) FV consumption | 0.108^***^ | 0.117^***^ | -0.132^***^ | -0.099^*^ | 0.309^***^ | 0.039 |
| (5) UPF consumption | 0.267^***^ | 0.058 | -0.127^***^ | 0.036 | 0.059 | 0.274^***^ |
| Study 2 |  |  |  |  |  |  |
| (1) Sleep quality | 0.065 | -0.126^***^ | -0.032 | -0.030 | 0.118^***^ | -0.251^***^ |
| (2) Physical activity | -0.064 | -0.088 | -0.090^**^ | 0.006 | 0.049 | -0.029 |
| (3) FV consumption | -0.036 | 0.131^***^ | -0.144^***^ | -0.071^*^ | 0.054 | -0.038 |
| (4) UPF consumption | -0.079^*^ | -0.054 | -0.013 | 0.121^***^ | 0.027 | 0.095^*^ |
| Study 3 |  |  |  |  |  |  |
| (1) Sleep quality, Raw | -0.035 | -0.064 | -0.003 | -0.043 | 0.164^*^ | -0.415^***^ |
| (2) Sleep quality, T | -0.026 | -0.052 | -0.016 | -0.046 | 0.153^*^ | -0.425^***^ |
| (3) Physical activity | 0.023 | -0.260^***^ | -0.006 | 0.102 | -0.053 | -0.043 |
| (4) Physical activity (F) | -0.158^*^ | -0.187^**^ | 0.024 | 0.048 | -0.018 | -0.102 |
| (5) FV consumption | -0.079 | 0.032 | -0.136^*^ | -0.104 | 0.147^*^ | -0.109 |
| (6) UPF consumption | -0.041 | 0.115 | -0.105 | -0.007 | 0.126 | 0.003 |

*p<.05, **p<.01, ***p<.001; BMI=body mass index; F=Fitbit measured; Raw=raw sleep quality scores; SES=socioeconomic status; T=sleep quality T scores. ^1^ male (0) vs. female/gender diverse (1). ^2^NZ European (0) vs non-NZ-European/mixed ethnicity (1). ^3^raw scores on depressive measures. Sleep quality values have been inversed for Study 1 and 3 so that higher values indicate greater sleep quality.

**SI Table 3***.* **Intra-class coefficient (ICC) values for the health behaviors and well-being measures in the daily diary studies.**

| Daily Measure | ICC (SE) |
| --- | --- |
| Study 2 |  |
| Sleep quality | 0.255 (0.012) |
| Physical activity | 0.269 (0.013) |
| FV consumption | 0.503 (0.014) |
| UPF consumption | 0.318 (0.014) |
| Well-being (raw) | 0.481 (0.014) |
| Study 3 |  |
| Sleep quality | 0.292 (0.029) |
| Physical activity | 0.367 (0.032) |
| Physical activity (Fitbit) | 0.282 (0.026) |
| FV consumption | 0.567 (0.029) |
| UPF consumption | 0.408 (0.031) |
| Well-being (raw) | 0.293 (0.026) |

FV=fruit and vegetables; ICC = intra-class correlation coefficient reflecting the percentage of variance attributable to between-person differences; SE=standard error; UPF=ultra-processed foods. The ICC represents the variance attributable to between-person differences (ICCs > .5 indicate more variance is between people; ICCs < .5 indicate more variance is within-people). Daily well-being was measured using the Flourishing Scale in Study 2 and the WHO-5 Well-Being Scale in Study 3 both adapted to daily reporting.

**SI Table 4***.* **A table comparing the outputs from Study 3’s models using the self-report measure of physical activity and Fitbit device measured physical activity .**

|  | Self-report model | Fitbit-measured model |
| --- | --- | --- |
| **Covariates** | *b* (SE), *p* | *b* (SE), *p* |
| Constant | 53.461 (2.396), <0.001 | 53.078 (2.433), <0.001 |
| Age | -0.446 (0.819), 0.586 | 0.078 (0.836), 0.925 |
| Gender |  |  |
| *Male* | (reference) |  |
| *Female* | -1.995 (2.328), 0.392 | -1.868 (2.322), 0.421 |
| *Gender diverse* | -19.526 (9.889), 0.048 | -19.23 (9.787), 0.049 |
| Ethnicity |  |  |
| *NZ European* | (reference) |  |
| *Māori* | 9.032 (10.041), 0.368 | 7.977 (9.888), 0.420 |
| *Asian* | -5.979 (4.315), 0.166 | -6.487 (4.32), 0.133 |
| *Indian* | 0.284 (6.817), 0.967 | -2.956 (6.802), 0.664 |
| *Other* | -3.408 (3.108), 0.273 | -4.028 (3.193), 0.207 |
| BMI | 0.308 (0.272), 0.258 | 0.318 (0.271), 0.241 |
| Childhood SES | 1.712 (0.846), 0.043 | 1.724 (0.859), 0.045 |
| Depressive symptoms |  |  |
| *Between-person* | -0.647 (0.132), <0.001 | -0.616 (0.132), <0.001 |
| *Within-person proxy* | -3.581 (0.226), <0.001 | -3.505 (0.231), <0.001 |
| **Health Behaviors** |  |  |
| *Between-person* |  |  |
| Sleep quantity | -0.8 (0.898), 0.373 | -0.608 (0.916), 0.507 |
| Sleep quality | 1.112 (0.236), <0.001 | 1.105 (0.237), <0.001 |
| Physical activity | 0.044 (0.027), 0.099 | 0.053 (0.027), 0.051 |
| FV consumption | 1.084 (0.594), 0.068 | 1.445 (0.589), 0.014 |
| UPF consumption | -1.072 (1.115), 0.336 | -1.43 (1.13), 0.206 |
| *Within-person* |  |  |
| Sleep quantity | 0.408 (0.306), 0.183 | 0.386 (0.31), 0.213 |
| Sleep quality | 0.483 (0.066), <0.001 | 0.46 (0.067), <0.001 |
| Physical activity | 0.040 (0.009), <0.001 | 0.035 (0.007), <0.001 |
| FV consumption | 0.957 (0.274), <0.001 | 0.893 (0.273), 0.001 |
| UPF consumption | 0.402 (0.416), 0.334 | 0.53 (0.426), 0.213 |
| AIC | 8721.84 | 8207.063 |
| BIC | 8841.384 | 8325.254 |

AIC=Aikake’s Information Criterion; BIC=Bayesian Information Criterion; FV= fruit and vegetable consumption; Quad=quadratic term; SES=socioeconomic status; UPF consumption= ultra-processed foods.

**SI Table 5. Results using health behaviors to predict well-being at the between-person and within-person levels, adjusting for covariates and allowing for two-way interactions between the health behaviors.**

|  | Study 1: Lifestyle of Young Adults (2021) | Study 2: Daily Life Study (2013-2014) | Study 3: Fitbit Study (2021-2022) |
| --- | --- | --- | --- |
| Outcome wellbeing | Flourishing (0-100%) | Flourishing (0-100%) | WHO-5 (0-100%) |
| n (participants) | 1,018 | 792 | 201 |
| k (responses) | 1,018 (1 per person) | 8,918 (~11.3 per person) | 1,024 (~5.1 per person) |
| Design | Cross-sectional | Daily diary | Daily diary |
| **Covariates** | *b* (SE), *p* | *b* (SE), *p* | *b* (SE), *p* |
| Constant | 70.262 (1.13), <0.001 | 61.938 (0.963), <0.001 | 53.285 (2.385), <0.001 |
| Age | -0.032 (0.188), 0.864 | -0.12 (0.233), 0.608 | -0.433 (0.816), 0.596 |
| Gender |  |  |  |
| *Male* | (reference) | (reference) | (reference) |
| *Female* | 4.19 (0.876), <0.001 | 2.359 (0.903), 0.009 | -1.811 (2.319), 0.435 |
| *Gender diverse* | 0.417 (4.128), 0.92 | - | -19.46 (9.846), 0.048 |
| Ethnicity |  |  |  |
| *NZ European* | (reference) | (reference) | (reference) |
| *Māori* | 3.183 (2.531), 0.209 | 2.111 (1.769), 0.233 | 11.221 (10.054), 0.264 |
| *Asian* | -3.015 (1.563), 0.054 | -3.907 (1.329), 0.003 | -5.694 (4.297), 0.185 |
| *Indian* | -0.025 (3.303), 0.994 | -4.74 (2.101), 0.024 | 0.445 (6.787), 0.948 |
| *Other* | -0.476 (1.324), 0.719 | 0.759 (2.485), 0.76 | -3.419 (3.094), 0.269 |
| BMI | -0.054 (0.079), 0.497 | -0.298 (0.093), 0.001 | 0.321 (0.271), 0.236 |
| Childhood SES | 2.032 (0.331), <0.001 | 0.347 (0.297), 0.242 |  |
| Depressive symptoms |  |  |  |
| *Between-person* | -0.943 (0.054), <0.001 | -0.421 (0.069), <0.001 | -0.657 (0.131), <0.001 |
| *Within-person proxy* | - | -3.441 (0.079), <0.001 | -3.554 (0.225), <0.001 |
| **Health Behaviors** |  |  |  |
| *Between-person* |  |  |  |
| Sleep quantity | 0.279 (0.158), 0.078 | -0.315 (0.485), 0.516 | -0.858 (0.895), 0.337 |
| Sleep quantity (quad) | - | -0.871 (0.345), 0.012 | - |
| Sleep quality | 0.185 (0.06), 0.002 | 8.591 (0.638), <0.001 | 1.106 (0.235), <0.001 |
| Sleep quality (quad) | -0.009 (0.004), 0.033 | - | - |
| Physical activity | 0.115 (0.025), <0.001 | 0.005 (0.018), 0.774 | 0.046 (0.027), 0.084 |
| Physical activity (quad) | -0.001 (0), 0.035 | - | - |
| FV consumption | 1.038 (0.204), <0.001 | 1.375 (0.338), <0.001 | 1.117 (0.592), 0.059 |
| FV consumption (quad) | -0.162 (0.052), 0.002 | -0.379 (0.159), 0.017 | - |
| UPF consumption | 1.022 (0.512), 0.046 | -1.284 (0.5), 0.01 | -0.985 (1.111), 0.375 |
| *Within-person* |  |  |  |
| Sleep quantity | n/a | -0.249 (0.094), 0.008 | 0.357 (0.304), 0.241 |
| Sleep quality | n/a | 2.173 (0.16), <0.001 | 0.463 (0.066), <0.001 |
| Physical activity | n/a | 0.038 (0.004), <0.001 | 0.037 (0.009), <0.001 |
| FV consumption | n/a | 0.536 (0.103), <0.001 | 1.092 (0.274), <0.001 |
| FV consumption (quad) | n/a | -0.135 (0.058), 0.021 | - |
| UPF consumption | n/a | 0.23 (0.122), 0.059 | 0.389 (0.414), 0.347 |
|  |  |  |  |
| *Continued below* |  |  |  |
|  |  |  |  |

| *Continued from above* |  |  |  |
| --- | --- | --- | --- |
| **Two-way Interactions** | Study 1: Lifestyle of Young Adults (2021) | Study 2: Daily Life Study (2013-2014) | Study 3: Fitbit Study (2021-2022) |
|  | *b* (SE), *p* | *b* (SE), *p* | *b* (SE), *p* |
| *Between x Between* |  |  |  |
| None significant | - | - | - |
| *Between x Within* |  |  |  |
| Sleep quality (b) x FV consumption (w) | n/a | -0.337 (0.158), 0.033 | - |
| Sleep quality (b) x UPF consumption (w) | n/a | 0.514 (0.183), 0.005 | - |
| Physical activity (b) x FV consumption (w) | n/a | - | 0.016 (0.006), 0.013 |
| *Within x Within* |  |  |  |
| Sleep quality x Physical activity | n/a | - | - |
| Sleep quality x Physical activity (quad) | n/a | - | - |
| Sleep quality x FV consumption | n/a | -0.186 (0.104), 0.074 | -0.161 (0.049), 0.001 |
| Sleep quality x FV consumption (quad) | n/a | 0.108 (0.051), 0.034 | - |
| AIC | 8288.643 | 71690.89 | 8710.496 |
| BIC | 8382.489 | 71904.02 | 8840.002 |

AIC=Aikake’s Information Criterion; (b)=between-person; BIC=Bayesian Information Criterion; FV=overall fruit and vegetable consumption; Quad=quadratic term; SES=socioeconomic status; UPF consumption= ultra-processed foods; (w)=within-person.

**SI Table 6***.* **A table comparing the outputs from Study 3’s two-way interaction models using the self-report measure of physical activity and Fitbit device measured physical activity.**

|  | Self-report model | Fitbit-measured model |
| --- | --- | --- |
| **Covariates** | *b* (SE), *p* | *b* (SE), *p* |
| Constant | 53.285 (2.385), <0.001 | 52.859 (2.426), <0.001 |
| Age | -0.433 (0.816), 0.596 | 0.087 (0.833), 0.917 |
| Gender |  |  |
| *Male* | (reference) | (reference) |
| *Female* | -1.811 (2.319), 0.435 | -1.65 (2.315), 0.476 |
| *Gender diverse* | -19.46 (9.846), 0.048 | -19.172 (9.757), 0.049 |
| Ethnicity |  |  |
| NZ European | (reference) | (reference) |
| Māori | 11.221 (10.054), 0.264 | 10.383 (9.909), 0.295 |
| Asian | -5.694 (4.297), 0.185 | -6.111 (4.308), 0.156 |
| Indian | 0.445 (6.787), 0.948 | -2.736 (6.78), 0.687 |
| Other | -3.419 (3.094), 0.269 | -3.964 (3.183), 0.213 |
| BMI | 0.321 (0.271), 0.236 | 0.333 (0.27), 0.217 |
| Childhood SES | 1.739 (0.842), 0.039 | 1.734 (0.856), 0.043 |
| Depressive symptoms |  |  |
| (continued below) |  |  |
| *Between-person* | -0.657 (0.131), <0.001 | -0.627 (0.132), <0.001 |
| *Within-person proxy* | -3.554 (0.225), <0.001 | -3.464 (0.23), <0.001 |
| **Health Behaviors** |  |  |
| *Between-person* |  |  |
| Sleep quantity | -0.858 (0.895), 0.337 | -0.684 (0.913), 0.454 |
| Sleep quality | 1.106 (0.235), <0.001 | 1.103 (0.237), <0.001 |
| *Continued below* |  |  |
| Physical activity | 0.046 (0.027), 0.084 | 0.053 (0.027), 0.048 |
| FV consumption | 1.117 (0.592), 0.059 | -1.312 (1.126), 0.244 |
| UPF consumption | -0.985 (1.111), 0.375 | 1.489 (0.588), 0.011 |
| *Within-person* |  |  |
| Sleep quantity | 0.357 (0.304), 0.241 | 0.328 (0.308), 0.287 |
| Sleep quality | 0.463 (0.066), <0.001 | 0.446 (0.066), <0.001 |
| Physical activity | 0.037 (0.009), <0.001 | 0.033 (0.007), <0.001 |
| FV consumption | 1.092 (0.274), <0.001 | 1.033 (0.273), <0.001 |
| UPF consumption | 0.389 (0.414), 0.347 | 0.468 (0.423), 0.268 |
|  |  |  |
| **Two-way Interactions** |  |  |
| *Between x Between* |  |  |
| None significant | - | - |
| *Between x Within* |  |  |
| Physical activity (b) x FV consumption (w) | 0.016 (0.006), 0.013 | 0.017 (0.006), 0.007 |
| *Within x Within* |  |  |
| Sleep quality x FV consumption | -0.161 (0.049), 0.001 | -0.153 (0.049), 0.002 |
| AIC | 8710.496 | 8195.761 |
| BIC | 8840.002 | 8323.801 |

AIC=Aikake’s Information Criterion; (b)=between-person; BIC=Bayesian Information Criterion; FV=overall fruit and vegetable consumption; Quad=quadratic term; SES=socioeconomic status; UPF consumption= ultra-processed foods; (w)=within-person.

**SI Table 7***.* **A comparison of each health behavior’s impact on the model’s R squared value when added first to the null model (covariates only).**

| Health Behavior | Study 1: Lifestyle of Young Adults (2021) | Study 2: Daily Life Study (2013-2014) | Study 3: Fitbit Study (2021-2022) |
| --- | --- | --- | --- |
|  | R2_w_, R2_b_, R2 Total | R2_w_, R2_b_, R2 Total | R2_w_, R2_b_, R2 Total |
| Sleep quality | n/a, +1.07%, +1.07% | +1.56%, +10.08%, +11.63% | +1.96%, +5.56%, +7.53% |
| Physical activity | n/a, +3.42%, +3.42% | +0.06%, +0.4%, +0.99% | +0.43%, +1.44%, +1.88% |
| FV consumption | n/a, +3.11%, +3.11% | +0.19%, +1.78%, +1.97% | +0.43%, +1.68%, +2.11% |
| UPF consumption | n/a, +0.47%, +0.47% | +0.16%, +0.81%, +0.96% | -0.01%^1^, +0.01%, +0.00% |
| Sleep quantity | n/a, +0.29%, +0.29% | +0.03%, +0.84%, +0.87% | +0.78%, -0.31%, +0.47% |

FV=overall fruit and vegetable consumption; R2=R squared; R2_w_=Total R squared explained by the predictors at the within-person level; R2_b_=Total R squared explained by the predictors at the between-person level; SES=Socioeconomic status; UPF consumption= ultra-processed foods. ^1^ R squared can occasionally decrease when adding predictors to multilevel models due to: (i) chance fluctuation, or; (ii) the new predictor being redundant to a predictor already in the model. See Snijders & Bosker (1999; 138) for more detail.
